# Synthesis and Assessment of AMPS-Based Copolymers Prepared via Electron-Beam Irradiation for Ionic Conductive Hydrogels

**DOI:** 10.3390/polym14132547

**Published:** 2022-06-22

**Authors:** Hyun-Su Seo, Jin-Young Bae, Kiok Kwon, Seunghan Shin

**Affiliations:** 1 Green Chemistry & Materials Group, Korea Institute of Industrial Technology (KITECH), Cheonan 31056, Chungnam, Korea; radiant@kitech.re.kr (H.-S.S.); kioks@kitech.re.kr (K.K.); 2 Department of Polymer Science and Engineering, Sungkyunkwan University, Suwon 16419, Gyeonggi, Korea; b521@skku.edu; 3 Division of Convergence Manufacturing System Engineering, University of Science & Technology (UST), Daejeon 34113, Korea

**Keywords:** ionic monomer, hydrogel, electron-beam irradiation, ionic conductivity, adhesion property

## Abstract

In this study, ionic conductive hydrogels were prepared with 2-acrylamido-2-methyl-1-propanesulfonic acid (AMPS). Acrylic acid (AA), acrylamide (AAm), and 2-hydroxyethyl acrylate (HEA) were used as comonomers to complement the adhesion properties and ion conductivity of AMPS hydrogels. Hydrogels were prepared by irradiating a 20 kGy dose of E-beam to the aqueous monomer solution. With the E-beam irradiation, the polymer chain growth and network formation simultaneously proceeded to form a three-dimensional network. The preferred reaction was determined by the type of comonomer, and the structure of the hydrogel was changed accordingly. When AA or AAm was used as a comonomer, polymer growth and crosslinking proceeded together, so a hydrogel with increased peel strength and tensile strength could be prepared. In particular, in the case of AA, it was possible to prepare a hydrogel with improved adhesion without sacrificing ionic conductivity. When the molar ratio of AA to AMPS was 3.18, the 90° peel strength of AMPS hydrogel increased from 171 to 428 g_f_/25 mm, and ionic conductivity slightly decreased, from 0.93 to 0.84 S/m. By copolymerisation with HEA, polymer growth was preferred compared with chain crosslinking, and a hydrogel with lower peel strength, swelling ratio, and ionic conductivity than the pristine AMPS hydrogel was obtained.

## 1. Introduction

Hydrogels are three-dimensional networks of hydrophilic polymer chains that absorb large amounts of water without dissolution. They can have diverse physical properties through various synthetic methods and a wide selection of hydrophilic polymers and monomers. In general, hydrogels absorb water at least 20% of their total weight, and superabsorbent hydrogels absorb more than 95%. Due to this hydrophilicity, they have widely been used in various biomedical applications such as medical dressings, drug delivery, and tissue engineering [1,2].

Recently, polyelectrolyte hydrogels have attracted much attention due to their unique properties of interacting with salt, solvents, and macromolecules. They have been used as critical components for electronic devices, tissue engineering scaffolding, coatings, fuel cells, water purification, drug delivery, etc. [3,4,5,6,7]. Polyelectrolyte hydrogels are generally prepared with ionic monomers [8], and among the ionic monomers, 2-acrylamido-2-methyl-1-propanesulfonic acid (AMPS) is selected due to its strongly ionizable sulfonate group [9,10,11]. In particular, AMPS hydrogels have been tested as patches for electronic devices such as electromyography (EMG) and electrocardiography (ECG) systems due to their stable ionic conductivity [12,13]. However, AMPS hydrogels have poor mechanical properties because of their low cohesive force. These shortcomings can be overcome by copolymerisation with hydrophilic or hydrophobic monomers and the addition of crosslinking agents.

A common method to prepare hydrogels from reactive monomers is to use thermal initiators. However, a thermal initiator requires a long reaction time and a high reaction temperature: Potassium persulfate requires more than 5 h at 40 °C for polymerisation [14]. A photopolymerisation method has been performed as an alternative, but it requires a photoinitiator that is irritating to the human body [15]. Although the reaction time is shortened compared with thermal polymerisation, photopolymerisation is delayed due to the moisture in the hydrogel and oxygen in the atmosphere [16]. Compared with these two conventional methods, an electron beam (E-beam) is a very effective source to produce hydrogels. E-beam radiation polymerisation is possible at room temperature in a short time without an initiator, and sterilisation is simultaneously performed [17,18]. Due to these advantages, E-beams have been applied to various materials from reactive monomers to various water-soluble polymers [19,20]. However, it is very difficult to analyse the structure of hydrogels because various active species are generated during the E-beam irradiation, which induces chain branching and crosslinking [21,22].

Extensive studies have been conducted to improve the cohesive energy of AMPS hydrogels through conventional radical copolymerisation with acrylic monomers such as acrylic acid (AA) and acrylamide (AAm) [23,24]. Their main concern was elastic modulus because AMPS hydrogels were mainly used as superabsorbent hydrogels. However, in order to use AMPS hydrogels as electronic device patches or motion sensor, their interfacial adhesion and ionic conductivity become more important for stable signal transmission.

Considering the fact that strong hydrogel adhesion depends on the synergy of the chemistry of bonds, topologies of connection, and mechanics of dissipation [25], the adhesion properties of AMPS hydrogels can be improved through copolymerisation with reactive monomers. In fact, it is difficult to obtain a high-molecular-weight linear polymer with an AMPS monomer due to the charge repulsion by its sulfonate group [26]. As a result, AMPS homopolymer has low cohesive energy. However, the molecular weight of AMPS polymer can be increased by copolymerisation with a comonomer that reduces the charge repulsion, and the cohesive energy increases accordingly. In addition, specific interactions such as H-bonding, electrostatic interactions, and hydrophilic and hydrophobic interactions also depend on the type of comonomer. Moreover, E-beam irradiation changes polymer chain structures and affects the physical properties of the hydrogel. E-beam copolymerisation is effective in improving the structure and physical properties of AMPS hydrogels, but there are a few related studies. In particular, to the best of our knowledge, there are few studies on the copolymerisation effect on the adhesive properties and ionic conductivity of AMPS hydrogels prepared via E-beam irradiation.

In this study, AMPS and AMPS/comonomer hydrogels were prepared through E-beam irradiation. Copolymerisation was attempted with representative hydrophilic monomers AA, AAm, and 2-hydroxyethyl acrylate (HEA) as comonomers to improve the adhesion properties and ionic conductivity of the AMPS hydrogel, which are required for electronic device patch application. The changes in mechanical and adhesion properties, water absorption, and ionic conductivity of AMPS hydrogels via copolymerisation were studied according to the type and content of the comonomer. In particular, the relationship between changes in the hydrogel structure and physical properties during E-beam irradiation was analysed with a focus on comonomers.

## 2. Experimental

### 2.1. Materials

**Figure 1:**
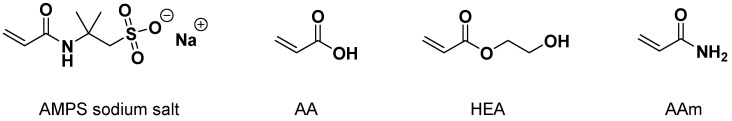
Chemical structures of AMPS sodium salt and comonomers.

2-Acrylamido-2-methylpropanesulphonic acid sodium salt solution (AMPS sodium salt, 50 wt% in H_2_O), acrylic acid (AA, 99%), 2-hydroxyethyl acrylate (HEA, 96%), and acrylamide (AAm, ≥98.0%) were used as monomers, and their chemical structures are given in Figure 1. Ethylene glycol (EG) was used to suppress water evaporation, sodium hydroxide solution (NaOH, 50% in H_2_O) was used as a pH adjuster, and polyethylene glycol diacrylate (PEGDA, *M_n_* = 250) was used as a crosslinker. All reagents were purchased from Sigma-Aldrich (St. Louis, MO, USA) and used without further purification.

### 2.2. Sample Preparation

Hydrogels were prepared as follows: First, AMPS 50 wt% solution, distilled water, comonomer, and additives (EG and PEGDA) were mixed; then, NaOH solution was added dropwise to adjust the pH to 5.0. The mixture was poured into a Petri dish (100 mm × 15 mm) to a thickness of 3 mm. Then, it was cured using an E-beam system (Mevex, Stittsville, ON, Canada) built by the Seoul Radiation Service (South Korea), with an intensity of 20 kGy. The compositions of the hydrogel samples are given in Table 1.

### 2.3. Measurements

The 90° peel strength of hydrogels was measured using a SurTA 2D peel tester (Chemilab, Gimpo, Gyeonggi, Korea). The hydrogel sample was cut into 25 mm × 60 mm rectangles with 3 ± 0.3 mm thickness and attached to the corona-treated PET film (0.1 mm thickness), using a Permabond 102 (Permabond Engineering Adhesives, Somerset, NJ, USA). It was attached to a glass substrate and left for 20 min. Afterwards, a 90° peel test was performed at a speed of 5 mm/s. At least five samples were measured.

The gel fraction of the dried hydrogel was calculated based on the Soxhlet extraction method. The hydrogel was placed in a vacuum oven at 60 °C for 24 h to remove water. Afterwards, the dried gel was weighed (*w*_1_), placed in the Soxhlet, and extracted using 100 °C water for 24 h. The extracted gel was dried in a vacuum at 60 °C for 24 h and subsequently weighed (*w*_2_). The gel fraction was calculated using the following equation:

$$\mathrm{Gel}\;\mathrm{fraction}\;\left(\%\right)=\frac{w_{2}}{w_{1}}\times 100$$

where *w*_1_ is the dried sample weight after water removal, and *w*_2_ is the dried sample weight after extraction.

The ionic conductivities of the hydrogels were measured via electrochemical impedance spectroscopy (EIS) using an Autolab PGSTAT 204 potentiostat (Metrohm, Herisau, Switzerland). The hydrogel samples, which were cut into 10 mm × 10 mm squares, were sandwiched between two ITO glasses. The EIS measurements were conducted under open-circuit conditions in the frequency range of 0.1–100 kHz, with an excitation voltage of 10 mV. The NOVA software (Version 2.1, Metrohm, Herisau, Switzerland) was used to fit the impedance data, and the bulk resistance (*R_o_*) was obtained as an x-intercept in the high-frequency region [27,28]. The obtained bulk resistance (*R_o_*) values were used to calculate the ionic conductivities of the hydrogels using the following equation:

$$\sigma =\frac{l}{R_{o}\times A}$$

where *σ* is the ionic conductivity, *R_o_* is the bulk resistance, *l* is the sample thickness, and *A* is the sample area. All measurements were repeated three times, and the average value was used.

The resistance change by the repeated deformation of the hydrogel was measured according to reference [29]. The hydrogel was cut into 15 mm × 45 mm rectangular pieces with a thickness of 2.1 mm. The copper wire was wound around both ends of a hydrogel and connected to a Digital Multimeter B45T (Owon, Zhangzhou, China). After the hydrogel was fixed on the index finger to detect the degree of finger bending, the resistance change was measured by repeatedly folding and unfolding the finger once every 3 s. During the repeated deformation, the original resistance and the resistance after folding were measured; then, the resistance change was calculated using the following equation:

$$\frac{\Delta R}{R_{0}}\left(\%\right)=\frac{R-R_{0}}{R_{0}}\times 100$$

where *R*_0_ is the resistance before folding, and *R* is the resistance after folding.

For the swelling ratio, 2 g of dried hydrogel square was placed in excess distilled water at room temperature. After preselected time intervals, the square was removed from the water, and its surface was wiped dry with a tissue. The swelling ratio was calculated as follows:

$$\mathrm{Swelling}\;\mathrm{ratio}\;\left(\%\right)=\frac{w_{t}-w_{0}}{w_{0}}\times 100$$

where *w*_0_ is the initial weight of the dried hydrogel, and *w_t_* is the weight of swollen hydrogel after a certain time.

Rheological measurements were performed in a rheometer MCR 102 (Anton Paar, Austria) using a 25 mm parallel-plate system. The hydrogel was cut into a disc with a 25 mm diameter and placed in the centre of the bottom plate. After installing the parallel plate, each sample was tested in the frequency sweep mode. The frequency sweep was 0.1–10 Hz, and the applied strain was set at 0.1%.

The porous structures of the hydrogels were observed via scanning electron microscopy (SEM). The hydrogel was cut into an appropriate size and immersed in deionised water for approximately 12 h to remove EG from the inside of the hydrogel sample [30]. Afterwards, it was cooled at −20 °C for 48 h and freeze-dried at −50 °C for 72 h in a freeze dryer (FDU-1200, EYELA, Japan). Thereafter, the lyophilised sample was sputtered for 30 s using a Sputter Coater 108auto (Cressington Scientific Instruments, Watford, UK) and characterised using a JSM-7601F SEM (JEOL, Tokyo, Japan).

In order to study the effect of the monomer on the formation of polymer chains and crosslinks, model hydrogels were prepared using a 5 kGy E-beam dose without PEGDA, and other compositions are shown in Table 1. The prepared hydrogel (0.5 g) was dissolved in 100 mL of 10 m urea solution for 15 days. The precipitate was filtered and then weighed after vacuum drying at 80 °C for 24 h. The polymer solution containing urea was poured into a dialysis tube (3.5–5 kD MWCO CE tubing, Biotech) and dialysed by changing the water 5–6 times for 3 days. To concentrate the solution, it was dialysed against PEG 10,000 for one more day. The supernatant solution was analysed using an aqueous gel permeation chromatography (water–GPC). The Thermo Dionex HPLC Ultimate 3000 (Dionex, Sunnyvale, CA, USA) was used with Waters Ultrahydrogel 120, 500, and 1000 columns. Sodium azide aqueous solution (0.1 M) was used as the eluent, and the flow rate was 1 mL/min. Pullulan and PEG standard solutions were used for calibration [31].

## 3. Results and Discussion

### 3.1. Polymer Structure of Hydrogels

Analysis of the crosslinking structure of hydrogels has been attempted by several research groups. Sen et al. reported a method to obtain the average molecular weight between crosslinks (${\bar{M}}_{c}$) from the swelling behaviours of diprotic acid-containing hydrogels [32]. However, their method is not applicable to a hydrogel prepared with an ionic monomer because they used the theoretical equation developed for nonionic networks. Meanwhile, Jovanovic et al. measured xerogel density via the pycnometry method and calculated molar mass between the network crosslinks using a nominal crosslinking ratio. They calculated the crosslinking density of xerogel and the distance between macromolecular chains using xerogel density and molar mass between the network crosslinks [33,34]. However, unlike thermal or UV radical polymerisation, E-beam polymerisation is likely to induce chain branching and crosslinking through hydrogen abstraction in a growing polymer chain. Therefore, the nominal crosslinking ratio calculated as the molar ratio of crosslinking agent (diacrylate) to monomer (monoacrylate) cannot be used in E-beam polymerisation.

**Figure 2:**
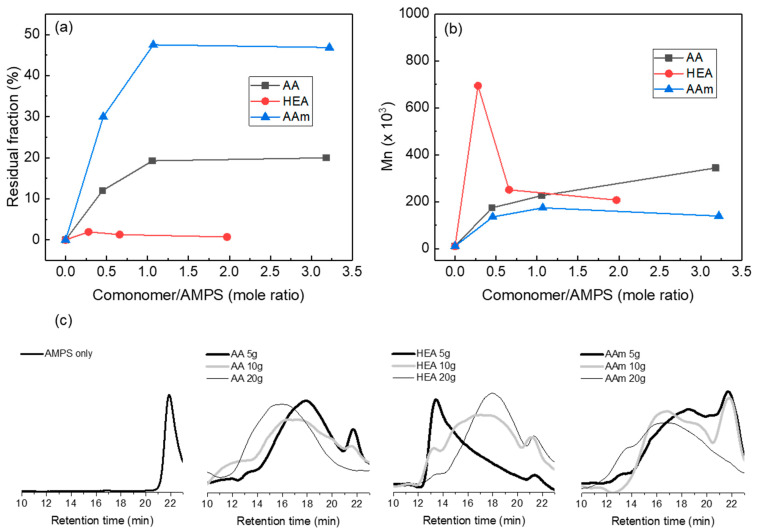
Residual fraction (%) of AMPS/comonomer hydrogels remained after extraction (**a**), number average molecular weight of the polymers isolated from AMPS/comonomer hydrogels (**b**), and AMPS/comonomer hydrogels water–GPC chromatograms (**c**). In this experiment, AMPS/comonomer hydrogels were prepared with a 5 kGy E-beam dose without PEGDA, and the number average molecular weight was calculated from the main peak in the water–GPC chromatograms.

Su et al. used an alternative method: They prepared a hydrogel with a low conversion using a thermal initiator, extracted a polymer solution from the hydrogel, and measured its molecular weight using water–GPC [31]. Therefore, we prepared model hydrogels, where chain branching and crosslinking were suppressed based on the composition in Table 1 while excluding PEGDA (crosslinking agent) and using a low E-beam dose (5 kGy). After the urea solution extraction, the model hydrogels gave different amounts of solid residues depending on the comonomer. Figure 2a shows that the residual gel fraction of a model hydrogel was almost 0% without a comonomer, but it increased to approximately 50% with AAm and 20% with AA, and less than 2% with HEA. Thus, AAm or AA can induce chain crosslinking even at a low E-beam dose, but chain crosslinking hardly occurs with HEA. This suggests that the hydrogen abstraction in a growing polymer chain occurred easily due to the radical stabilisation by the pendant group of AAm or AA.

In order to estimate the molecular weight of the polymer chains prepared using an E-beam, the molecular weight of the extracted polymer was measured. Figure 2b shows the molecular weights of the extracted polymers calculated from the highest peak in the water–GPC chromatograms. As shown in Figure 2c, water–GPC chromatograms are very broad because branched polymers can be included in the extracted polymers. Although the measured molecular weight may be inaccurate, this method can provide approximate information about the polymer size produced via E-beam polymerisation.

In the water–GPC chromatograms, the AMPS homopolymer was observed at a retention time of 22 min, and its average molecular weight was 10.5 Kg/mol. In general, when radical copolymerisation was performed with two different monomers, homo- or copolymerisation competitively occurred, depending on the relative reactivity and amounts of the monomers. Figure 2c also shows that when the amount of AAm or AA increased, the peak assigned to the AMPS homopolymer decreased, but the peak assigned to the AMPS-*co*-AAm or -AA copolymer increased and shifted to a shorter retention time (high molecular weight). In copolymerisation with AAm, 134 K, 174 K, and 139 Kg/mol copolymers appeared when the AAm content increased, but the intensity of the AMPS homopolymer decreased. In copolymerisation with AA, 173 K, 226 K, and 344 Kg/mol copolymers were observed with increasing AA content. Meanwhile, AMPS-*co*-HEA hydrogels showed different results. When the HEA content increased, the molecular weight of the copolymer decreased to 690 K, 251 K, and 207 Kg/mol. When the HEA content was small (HEA/AMPS = 0.28), HEA homopolymerisation appeared to be preferred over copolymerisation. When the HEA content increased, the AMPS-*co*-HEA copolymer appeared, but the AMPS homopolymer was still observed at HEA/AMPS = 1.97. Combining the residual fraction and copolymer molecular weight results, AAm and AA facilitate both the growth of polymer chains and chain crosslinking, whereas HEA favours the growth of polymer chains over chain crosslinking.

### 3.2. Adhesion Properties of Hydrogels

AA, HEA, and AAm were added as comonomers to modify the adhesion properties of the AMPS hydrogel. The total amount of monomers (AMPS and comonomer) was fixed at 40 g, and the amount of comonomer was changed by 5, 10, and 20 g. Table 2 shows the molar ratio of comonomer to AMPS. Since AA and AAm have similar molecular weights, their molar ratios to AMPS were similar, but HEA showed a lower molar ratio due to its slightly higher molecular weight.

**Figure 3:**
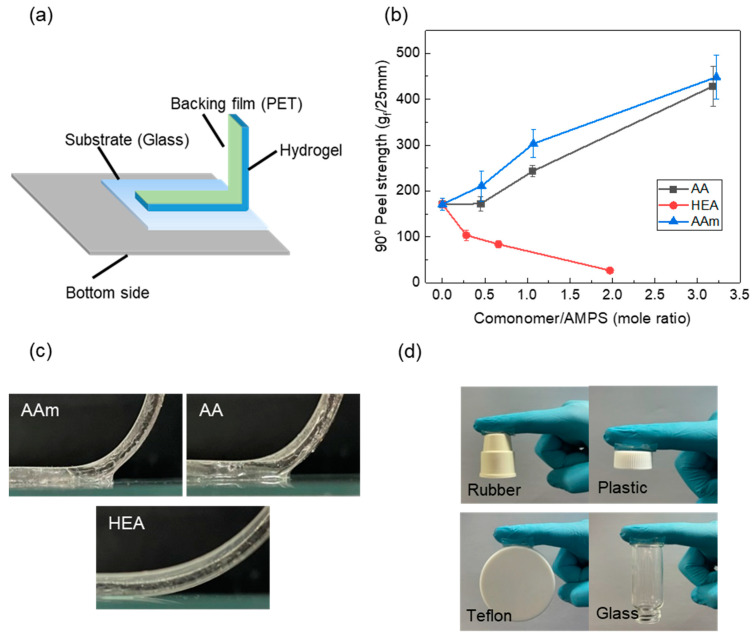
(**a**) Schematic diagram of the 90° peel test; (**b**) the 90° peel strength of AMPS hydrogels modified with different comonomers; (**c**) photos of the hydrogel–glass interface during peeling test; (**d**) adhesion of the AAm20 hydrogel on different materials.

The adhesion strength of hydrogels was measured using a 90° peel test, and the results are given in Figure 3. The 90° peel strength of the AMPS hydrogel increased due to the copolymerisation with AA or AAm, whereas it decreased with HEA. All hydrogels showed interfacial failure, which implies that the 90° peel strength of the hydrogel was determined by the interfacial adhesion strength. Due to the H-bonding between AA or AAm and a glass substrate, the interfacial strength of the AMPS hydrogel monotonically increased with the AA or AAm content. However, in the case of HEA, the 90° peel strength of the AMPS hydrogel decreased due to copolymerisation. Thus, the adhesion strength decreased due to the increase in cohesive force by copolymerisation, which seemed to surpass the increase in adhesive strength by H-bonding with HEA (Figure 3c).

**Figure 4:**
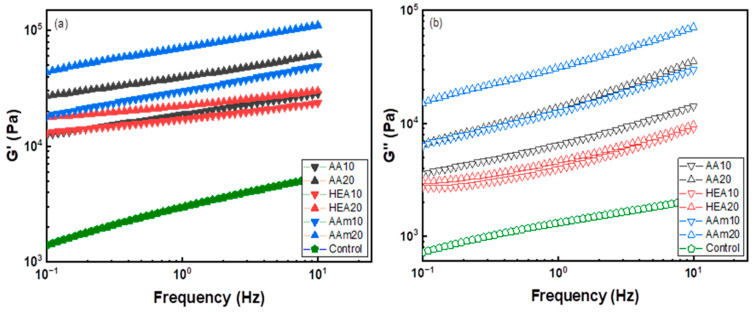
Storage (**a**) and loss modulus (**b**) of AMPS hydrogels modified with different comonomers.

The storage modulus of a hydrogel, which is strongly correlated with the cohesive force, was measured at room temperature using the small amplitude oscillatory shear (SAOS) method. Figure 4 shows that the storage modulus (G’) was higher than the loss modulus (G”), which implies that the elastic behaviour of a hydrogel dominates the physical properties. The storage modulus of the AMPS hydrogel remarkably increased due to the copolymerisation with a comonomer mainly because the comonomer alleviated the charge repulsion between AMPS and resulted in a high-molecular-weight AMPS copolymer. It is well known that charge repulsion between sulfonates of AMPS can interfere with the polymerisation and interchain crosslinking reactions of AMPS [9,35,36].

For the effect of each comonomer, the storage modulus of the AMPS hydrogel increased in the order of AAm > AA > HEA. G’ of AMPS hydrogels significantly increased with the AAm content but slightly increased with the HEA content. Since G’ and G” of polymers depend on the polymer chain size and interchain interactions such as chemical and physical crosslinks [37], it is suggested that AAm or AA increases the degree of polymerisation and induces strong interchain interactions.

**Figure 5:**
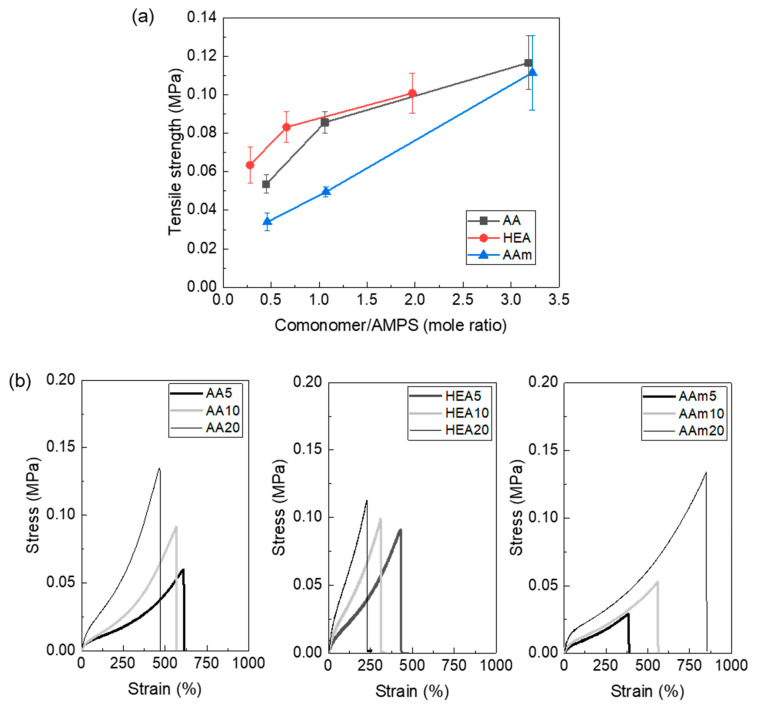
Tensile strength (**a**) and stress–strain curves (**b**) of AMPS hydrogels modified with different comonomers.

The tensile strength of the AMPS hydrogel also increased with copolymerisation (Figure 5a) because its cohesive force is increased by copolymerisation, as previously discussed. In this figure, the tensile strength of the AMPS hydrogel was not measured because it was too crushable to withstand the pressure of the tensile grip. The stress–strain curves (Figure 5b) showed that AMPS hydrogels copolymerised with AAm increased both stress and strain, which resulted in very tough hydrogels. To explain the comonomer effect on the polymer chain size and interchain interaction of the AMPS hydrogel, the hydrogel structures must be analysed.

### 3.3. Gel Fraction of Hydrogels

**Figure 6:**
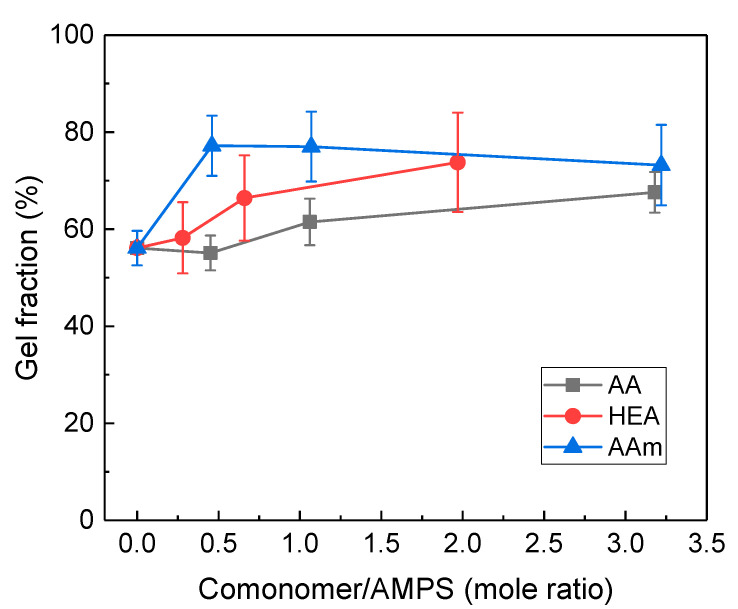
Gel fraction (%) of AMPS hydrogels modified with different comonomers: AA, HEA, and AAm.

The gel fraction of AMPS/comonomer hydrogels was measured using Soxhlet extraction, and the results are shown in Figure 6. The gel fraction was relatively higher than the residual fraction due to the difference in E-beam dose (5 kGy vs. 20 kGy) and PEGDA. In particular, the AMPS/HEA hydrogels showed a significantly higher gel fraction than the residual fraction. In Figure 2a, AMPS/HEA model samples (without PEGDA) showed a very small residual fraction. Thus, the crosslinking via copolymerisation with HEA was negligible, implying that the interaction between AMPS-*co*-HEA chains is relatively weak due to the increased free volume by the hydroxyethyl group of HEA [31].

**Figure 7:**
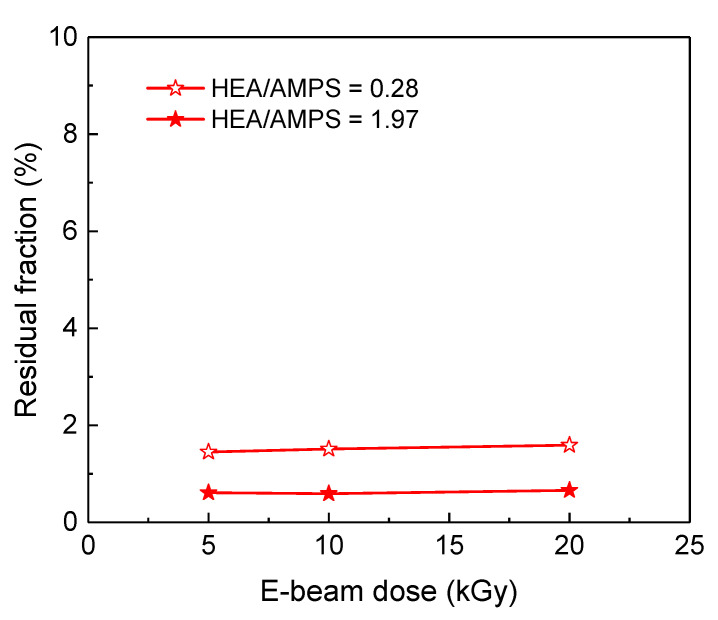
Residual fraction of model HEA-co-AMPS hydrogels as a function of the E-beam dose.

For the model HEA-*co*-AMPS hydrogel, its residual fraction was almost unchanged after the E-beam dose increased to 20 kGy (see Figure 7), which suggests that the increase in E-beam dose up to 20 kGy is not the main cause of the increase in gel fraction. Moreover, considering that the storage moduli of the AMPS/HEA hydrogels did not noticeably change with the HEA content (Figure 4a), there is little difference in chain interaction and crosslinking density due to HEA. Thus, the gel fraction of AMPS/HEA hydrogels mainly increased due to the addition of PEGDA. Even after the Soxhlet extraction with H_2_O vapour, unreacted monomers and linear copolymers might remain in the network structure by PEGDA; consequently, it is believed that a high gel fraction was measured.

### 3.4. Morphologies of Hydrogels

**Figure 8:**
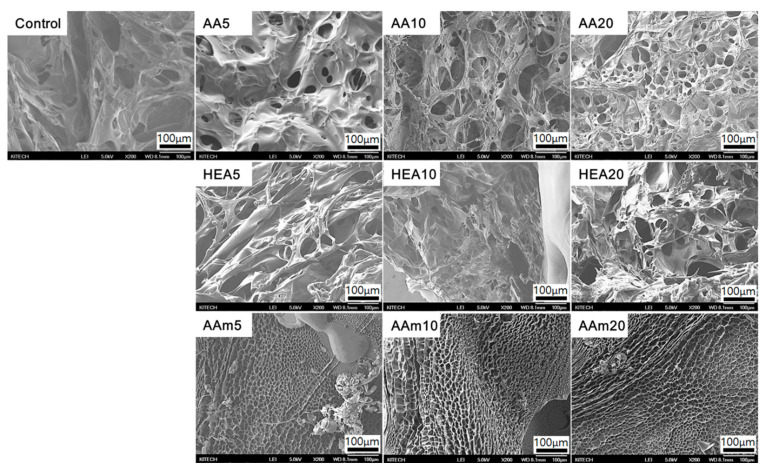
SEM images of fracture surfaces of AMPS hydrogels modified with different comonomers; magnification: ×200.

Hydrogel structures were analysed via FE-SEM to determine the effect of a comonomer on the pore structure of hydrogels. As displayed in Figure 8, all hydrogels had a porous structure, and their pore sizes varied with the type and amount of a comonomer. In particular, hydrogels prepared with AA as a comonomer showed many small pores and became more porous as the AA content increased. Meanwhile, there was no appreciable difference in the porous structures of HEA-modified hydrogels according to the comonomer content. In particular, AAm-modified hydrogels showed different fracture surfaces, and they had smaller and denser pore structures than the control, AA-modified, and HEA-modified hydrogels. The reason is that the AMPS/AAm hydrogels have high chemical and physical crosslinks due to the addition of AAm, as previously mentioned.

### 3.5. Swelling Ratio of Hydrogels

**Figure 9:**
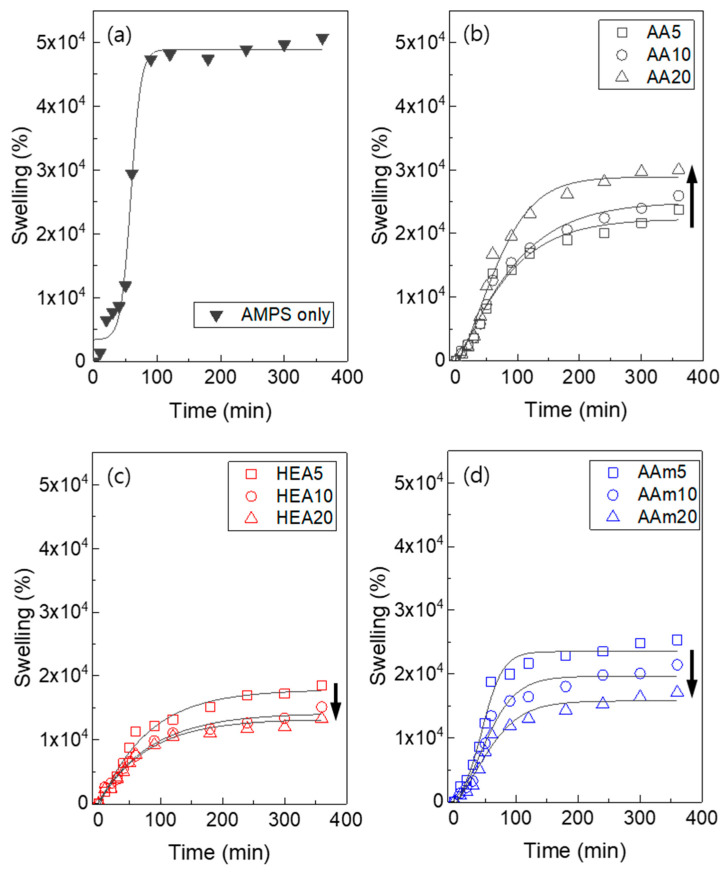
Swelling ratio of control AMPS hydrogel (**a**) and AMPS hydrogels modified with AA (**b**), HEA (**c**), and AAm (**d**).

The water absorption of a hydrogel is mainly dependent on the hydrophilicity of the monomer and the degree of crosslinking of the hydrogel. In particular, AMPS hydrogels are known to have high water absorption properties because AMPS is an ionic and very hydrophilic monomer [38]. Therefore, it is simply expected that the swelling ratios of AMPS/comonomer hydrogels will decrease with comonomer addition because AMPS is more hydrophilic than comonomers (AA, HEA, and AAm). In addition, from the results of Figure 2a and Figure 6, it can be inferred that the degree of crosslinking of the hydrogel also increased due to the addition of a comonomer, which decreased the swelling ratio of the hydrogel. Consequently, the AMPS/comonomer hydrogels showed a lower swelling ratio than the AMPS hydrogel (Figure 9).

Considering the effect of the comonomer type, HEA- or AAm-modified AMPS hydrogels showed that their swelling ratio simply decreased when the comonomer content increased. However, AA-modified AMPS hydrogels showed an increased swelling ratio with increasing AA content. These results are due to the differences in hydrophilicity of comonomers and pore structure. AA is an ionic monomer and has better water absorption properties than HEA or AAm. As presented in Figure 8, HEA- or AAm-modified AMPS hydrogels showed similar SEM images regardless of the comonomer content. Therefore, the swelling ratio simply decreased when the comonomer content increased. However, AA-modified AMPS hydrogels showed a more porous structure when the AA content increased, so it is speculated that the swelling ratio increased even if the AMPS content decreased.

### 3.6. Ionic Conductivity of Hydrogels

**Figure 10:**
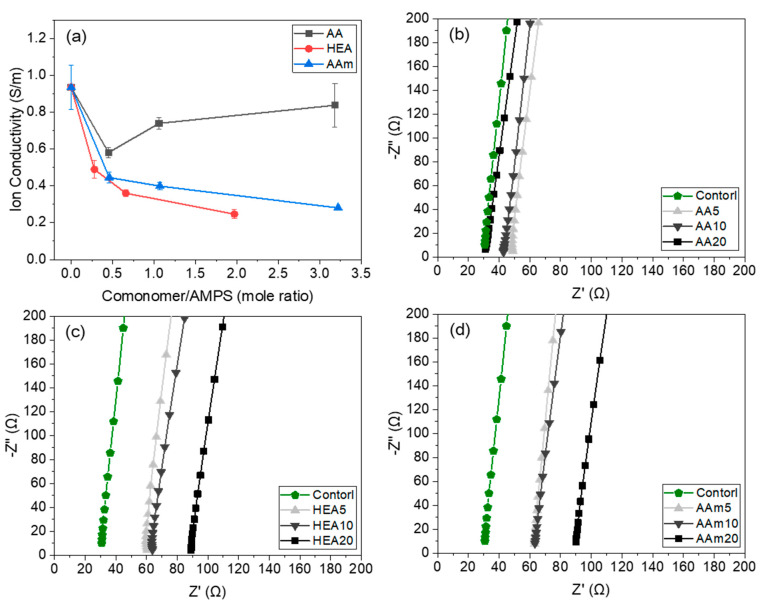
Ionic conductivity of AMPS hydrogels modified with different comonomers (**a**) and the partial enlarge plots of their electrochemical impedance spectroscopy (**b**–**d**).

Figure 10 shows the ionic conductivity of AMPS hydrogels modified with different comonomers and the partial enlarge plots of their electrochemical impedance spectroscopy. In this study, Na^+^ and Cl^−^ ions in the hydrogel are involved in charge transfer. The hydrophilicity and pore structure of AMPS/comonomer hydrogels depend on the type and amount of comonomer, and these differences alter the conductivity of Na^+^ and Cl^−^ ions in the hydrogel. Since HEA and AAm are nonionic monomers, the ionic conductivity of the HEA- or AAm-modified AMPS hydrogels decreased with increasing HEA or AAm content. However, AA, which is an anionic monomer, increased the ionic conductivity of the hydrogels. When 5 g of AA (mole ratio of AA to AMPS is 0.45 (AA/AMPS = 0.45)) was used, the hydrogel showed lower ionic conductivity than the AMPS hydrogel (0.58 S/m vs. 0.93 S/m) because AMPS has better ion conductivity than AA. However, when the AA content increased, the ionic conductivity of the hydrogel increased, and with 20 g of AA (AA/AMPS = 3.18), it approached the value of the AMPS hydrogel (0.84 S/m vs. 0.93 S/m), since AA induced many small pores in the hydrogel, as shown in Figure 8, and these pores could facilitate ions to diffuse across the hydrogel.

**Figure 11:**
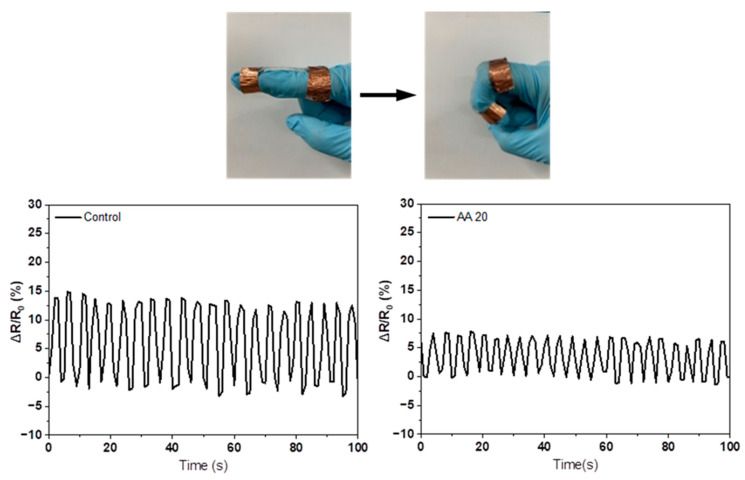
Change in resistance of the control and AA20 hydrogels.

To determine the changes in ionic conductivity due to the deformation of the hydrogel, the AMPS hydrogel and AA20 hydrogel were selected, and their resistance was measured by folding and unfolding the finger. Figure 11 shows that the AMPS hydrogel had 15% increased resistance by finger folding and returned to zero after finger unfolding. The reason is that the resistance of the hydrogel increased by narrowing the porous microstructure due to the bending of the finger [29]. Even after many repeated deformations, the AMPS hydrogel maintained its initial resistance. Meanwhile, the AA20 hydrogel showed that the resistance changed by less than 8%, which was a small change in resistance. Thus, for use as a conductive patch for devices such as EMG and ECG systems, the AA20 hydrogel is more advantageous than the AMPS hydrogel because it shows stable ionic conductivity against external deformation. EMG and ECG systems that detect the human body’s electrical signals are usually attached to the body using acrylic adhesives. Although AMPS-based hydrogels have superior ionic conductivity compared with acrylic adhesives, their adhesion strength is relatively low. Therefore, it is necessary to improve the adhesion strength of AMPS-based hydrogels to the level of acrylic adhesives. In addition, the study of maintaining adhesion on human skin during perspiration should be conducted in a future study.

## 4. Conclusions

In this study, ionic conductive hydrogels were prepared with AMPS using E-beam irradiation. To modify the properties of the AMPS hydrogel, representative acrylates such as AA, HEA, and AAm were used as comonomers, and the following conclusions were obtained:

AMPS copolymers showed significantly increased molecular weight, compared with AMPS homopolymers, irrespective of the type of comonomer. During E-beam copolymerisation, chain branching or crosslinking via hydrogen abstraction occurred in the growing polymer chain, and the chain crosslinking increased in the order of AAm > AA >> HEA. This suggested that AAm and AA induced chain crosslinking, as well as the growth of polymer chains, whereas HEA preferred the growth of polymer chains to chain crosslinking.

AA and AAm significantly increased the peel strength of the AMPS hydrogel through the enhanced cohesive force of the hydrogel and interfacial hydrogen bonding with a substrate. The AA20 hydrogel exhibited a 90° peel strength of 428 g_f_/25 mm, which was more than twice that of the AMPS hydrogel (171 g_f_/25 mm). However, HEA decreased the peel strength of the AMPS hydrogel due to its relatively low hydrogen bonding ability. 

The ionic conductivity of AMPS/comonomer hydrogels was affected by the hydrophilicity and pore structure of the hydrogel, which depended on the type and content of the comonomer. The AA20 hydrogel showed comparable ionic conductivity to AMPS hydrogel (0.84 S/m vs. 0.93 S/m) and less change in resistance to external deformation (8% vs. 15%). Unlike other comonomers, the swelling ratio of the AMPS/AA hydrogel increased with AA content, and the ionic conductivity became similar to that of the AMPS hydrogel. The reason is that AA has a high water affinity and induces many ion-channel pores in the hydrogel structure.

## Figures and Tables

**Table 1:** Compositions of AMPS hydrogels modified with comonomers.

Sample Code	AMPS Salt Solution (g)	AA (g)	HEA (g)	AAm (g)	H_2_O (g)	EG (g)	PEGDA (g)	NaOH
**Control**	80 (40/40)	0	0	0	0	20	0.08	pH = 5.0
**AA5**	70 (35/35)	5	0	0	5	20		
**AA10**	60 (30/30)	10	0	0	10	20		
**AA20**	40 (20/20)	20	0	0	20	20		
**HEA5**	70 (35/35)	0	5	0	5	20		
**HEA10**	60 (30/30)	0	10	0	10	20		
**HEA20**	40 (20/20)	0	20	0	20	20		
**AAm5**	70 (35/35)	0	0	5	5	20		
**AAm10**	60 (30/30)	0	0	10	10	20		
**AAm20**	40 (20/20)	0	0	20	20	20		

**Table 2:** Molar ratio of comonomer to AMPS in each sample.

Sample Code	AA5	AA10	AA20	HEA5	HEA10	HEA20	AAm5	AAm10	AAm20
Comonomer/AMPS (mole ratio)	0.45	1.06	3.18	0.28	0.66	1.97	0.46	1.07	3.22

## Data Availability

The data presented in this study are available on request from the corresponding author.
